# Hierarchical Porous Molybdenum Carbide Based Nanomaterials for Electrocatalytic Hydrogen Production

**DOI:** 10.3389/fchem.2020.00426

**Published:** 2020-05-19

**Authors:** Yan Liu, Juanjuan Huo, Jiaojiao Guo, Li Lu, Ziyan Shen, Weihua Chen, Chuntai Liu, Hao Liu

**Affiliations:** ^1^Joint International Laboratory on Environmental and Energy Frontier Materials, School of Environmental and Chemical Engineering, Shanghai University, Shanghai, China; ^2^Key Laboratory of Materials Processing and Mold (Zhengzhou University), Ministry of Education, Zhengzhou, China; ^3^Centre for Clean Energy Technology, School of Mathematical and Physical Sciences, Faculty of Science, University of Technology Sydney, Sydney, NSW, Australia

**Keywords:** hierarchical structure, electrocatalytic reaction, hydrogen production, porous structure, molybdenum carbide

## Abstract

The electrocatalytic hydrogen evolution reaction (HER) for the preparation of hydrogen fuel is a very promising technology to solve the shortage of hydrogen storage. However, in practical applications, HER catalysts with excellent performance and moderate price are very rare. Molybdenum carbide (Mo_x_C) has attracted extensive attention due to its electronic structure and natural abundance. Here, a comprehensive review of the preparation and performance control of hierarchical porous molybdenum carbide (HP-Mo_x_C) based catalysts is summarized. The methods for preparing hierarchical porous materials and the regulation of their HER performance are mainly described. Briefly, the HP-Mo_x_C based catalysts were prepared by template method, morphology-conserved transformations method, and secondary conversion method of an organic-inorganic hybrid material. The intrinsic HER kinetics are enhanced by the introduction of a carbon-based support, heteroatom doping, and the construction of a heterostructure. Finally, the future development of HP-Mo_x_C based catalysts is prospected in this review.

## Introduction

Hydrogen is a green energy with high energy density and excellent combustion performance (Martinez et al., 2019; Yang et al., 2019). HER is a key reaction for the renewable production of hydrogen. However, the actual reaction process is inefficient. In order to increase the conversion efficiency of the reaction process and reduce the reaction overpotential, a certain amount of catalyst is usually used (Chen et al., 2018; Huo et al., 2019; Ling et al., 2019). The ideal electrocatalyst for the HER is platinum (Pt) or other precious metals, but its application is severely limited by low richness and high cost (Khaselev and Turner, 1998; Nong et al., 2018).

Mo_x_C has a wide range of applications in the fields of energy storage and conversion, for example, hydrodesulf‘urization, denitrification (Wang et al., 2007; Ma et al., 2018), methanol reforming, electrolyte, etc. (Gao et al., 2010; Lin et al., 2017b; Yang et al., 2020). Density functional theory (DFT) calculations of carbides show that the hybridization of metal d orbitals with carbon s and p orbitals causes wider d-band structure, showing a d-band structure similar to Pt (Zhao et al., 2019b). This makes Mo_x_C a promising alternative to precious metal catalysts. Conventional Mo_x_C based catalysts generally have no voids or low porosity, resulting in low active surface area and poor wettability. Designing hierarchical porous micro/nanostructures can solve these problems. The hierarchical porous material has multi-stage pore structure, which is micropores (<2 nm), mesopores (2–50 nm) and macropores (>50 nm) (Li et al., 2019b). The properties and functions of a material depend on the characteristics of its structure, such as pore size, shape, porosity, etc. (Ryoo, 2019). In general, the presence of micropores provides a large surface area, mesoporous, and macroporous structures are effective in improving electrolyte penetration and promoting ion diffusion. The structure of hierarchical porous materials is usually assembled from nanoscale units by van der Waals forces, ionic bonds, covalent bonds and hydrogen bonds. The preparation of HP-Mo_x_C based catalysts prevents the agglomeration of the nanoparticles, greatly increasing the specific surface area of the material and exposing more active sites (Kim et al., 2020). Compared with other non-precious metal catalysts, HP-Mo_x_C has a hierarchical porous structure on the macro scale and a d-band structure similar to Pt on the micro scale, which makes it exhibiting unique advantages.

This review focuses on the preparation and performance of HP-Mo_x_C based catalysts, including soft-hard template method, morphology-conserved transformations, secondary conversion of organic-inorganic hybrid materials to construct catalysts with specific morphology. By introducing other conductive carriers, heterogeneous doping and construct heterostructured hybrids to optimize the HER performance of HP-Mo_x_C based catalysts. Table 1 shows the performance parameters of each catalyst mentioned in this article. Finally, an overview of the future development of HP-Mo_x_C based electrocatalysts is outlined.

**Table 1:** Summary of HER performance of Pt/C and various catalysts appearing in the article.

	**Method**	**Catalyst**	**η_onset_ (mv)**	**η_10_ (mv)**	**Electrolyte**	**Tafel slope (mv dec^**−1**^)**	**References**
Preparation	Template method	uf-Mo_2_C/CF	49	184	Acidic	71	Kou et al., 2018
		Mo_2_C/MCS	73	134	Alkaline	51	Yuan et al., 2019
	Morphology-conserved transformations	Nano MoC@GS	84	132	Acidic	46	Shi et al., 2016
		Porous MoCx nano-octahedrons	25	142	Acidic	5	Wu et al., 2015
			80	151	Alkaline	59	
		MoC-Mo_2_C/PNCDS	121	\	Alkaline	60	Lu et al., 2019
	Secondary conversion of organic-inorganic hybrid materials	np-Mo_2_C NW	70	\	Acidic	\	Liao et al., 2014
		P-Mo_2_C NWs	42	89	Acidic	42	Shi et al., 2017
Regulation	Introducing other conductive carriers	Mo_2_C-RGO	70	130	Acidic	54	Pan et al., 2014
		Mo_2_C/G	\	175	Acidic	88	Huang et al., 2019c
			\	200	Alkaline	82	
		Mo_2_C@NC nanomesh	\	37.5	Acidic	33.7	Cheng et al., 2018
	Doping	Mo_2_C-N-CNFS	105	192	Acidic	70	Wu et al., 2016
		Ni/Mo_2_C-NCNFS	29	143	Alkaline	57.8	Li et al., 2019b
		NP-MO_2_C	\	210	Acidic	64	Wang et al., 2018a
	Hierarchical porous molybdenum carbide-based heterostructure	Mo-Mo_2_C	67	150	Acidic	55	Dong et al., 2018
		Mo_2_C/VC@C	\	122	Acidic	43.8	Huang et al., 2019a
		Pt/C	0	28	Acidic	33	
			0	43	Alkaline	113	

*η_10_, overpotentials to drive the current densities of 10 mA cm^−2^*.

*η_onset_, oneset overpotential*.

## Construction of HP-Mo_x_C With Special Morphology

HP-Mo_x_C based catalysts with a special morphology have many excellent properties such as rapid mass transfer, ultra-high surface area, controlled pore size and nano-effects (Niu et al., 2019; Wang et al., 2019b). Therefore, it is becoming more and more important to construct various forms of nano-catalytic materials. The use of nanotechnology makes it possible to expose as many active sites as possible during electrocatalysis, thereby improving HER performance (Hou et al., 2019). However, how to control the structural size and shape of materials still poses great challenges in the current research process.

### Template Method to Construct HP-Mo_x_C Based Catalysts

The template method is one of the effective methods for preparing hierarchical porous materials, and can effectively control the morphology, particle size, and structure during the preparation process (Huang et al., 2019b; Zhao et al., 2019a). It is mainly divided into hard template method and soft template method. The hard template has rigid structure and specific morphology, and its morphology is copied into the target material by nano-replication technology. The obtained product has good dispersibility, controllable pore size and has been widely used (Chen et al., 2019; Feng et al., 2019).

**Figure 1:**
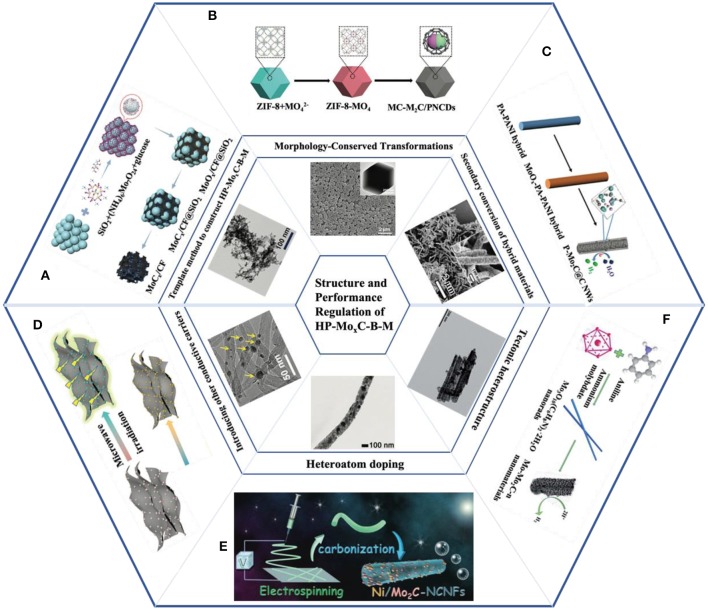
Schematic illustration of synthesis and structures of various HP-Mo_x_C based catalysts: **(A)** Schematic synthesis route and TEM image of the uf-Mo_2_C/C. Reprinted with permission from Kou et al. (2018) with permission from WILEY-VCH. **(B)** Schematic synthesis route and SEM/TEM images of the MoC-Mo_2_C/PNCDs. Reprinted with permission from Lu et al. (2019) with permission from WILEY-VCH. **(C)** Schematic synthesis route and SEM image of the P-Mo_2_C@C NWs. Reprinted with permission from Shi et al. (2017) with permission from Royal Society of Chemistry. **(D)** Schematic synthesis route and HR-TEM image of the Mo_2_C/G. Reprinted with permission from Huang et al. (2019c) with permission from WILEY-VCH. **(E)** Schematic synthesis route and TEM image of the Ni/Mo_2_C-NCNFs. Reprinted with permission from Li et al. (2019b) with permission from WILEY-VCH. **(F)** Schematic synthesis route and TEM image of the Mo-Mo_2_C. Reprinted with permission from Dong et al. (2018) with permission from Royal Society of Chemistry.

Due to the stability of the hard template structure, the precursors are often used as “microreactor” in the synthesis process (Liu et al., 2019). The colloidal crystal (Thompson et al., 2019) contains a large amount of monodisperse colloidal particles, which are uniformly arranged in three dimensions. Using colloidal crystals as sacrificial hard templates, ordered and monodisperse pores can be introduced into the material. Kou et al. (2018) prepared hierarchical porous molybdenum carbide nanocrystals (uf-Mo_2_C/CF) with efficient HER performance by using uniformly-sized SiO_2_ microspheres as confined template (Figure 1A). Average size of nanocrystals is <2 nm. This 3D hierarchical porous structure enables a large number of mass transfer channels, high density of active sites, and high electrical conductivity, thereby providing a highly efficient and stable catalytic performance. Soft template method has no fixed structure and morphology. Soft templating agent mainly forms an organic phase with a certain morphology through intermolecular or intramolecular interaction forces (Xue et al., 2019; Zhang et al., 2019). In the process of synthesis, soft templating agent interacts with the inorganic phase to form an organic-inorganic phase with a certain morphology, thereby achieving the purpose of directional synthesis of nanomaterials. Yuan et al. (2019) used the precursor spheres formed by F127 and resoled phenolic resin to limit the growth of molybdenum carbide, and obtained ultra-small Mo_2_C particles encapsulated *in situ* in mesoporous carbon spheres (Mo_2_C@MCS). The ultra-small particle size exposes more active sites, and the presence of a carbon substrate greatly reduces the resistance of the catalyst, thereby exhibiting excellent electrocatalytic performance in an alkaline medium (Liu et al., 2018; Huang et al., 2019d). This study provides an effective strategy for the synthesis of a Mo_x_C@C catalyst.

### Morphology-Conserved Transformations

Although the template method can effectively control the morphology and pore size of the catalyst, the synthesis and the removal process of the template cause a lot of waste of resources and increase in cost, which hinders its application. The morphology-conserved transformations method has been developed as a simple and effective synthesis route (Tan et al., 2012). This method usually consists of two steps: first, a hierarchical porous metal intermediate compound is constructed and then converted into a carbon material by specific method. Metal organic frame materials (MOF) have become one of the most promising candidate precursors for the preparation of hierarchical porous materials due to their advantages such as uniform and controllable pore structure and large specific surface area (Dhakshinamoorthy et al., 2019; Garzón-Tovar et al., 2019). Shi et al. (2016) synthesized a highly active and stable MoC encapsulated by graphitized carbon shell (nanoMoC@GS) electrocatalyst by *in-situ* carburization of Mo-based MOF, achieving “atomic-level contact” between Mo and organic species. The rich organic matter in the MOF generates a porous conductive carbon shell layer, which improves the ion transmission to the medium. Wu et al. (2015) adopted the “MOFs-assisted synthesis strategy” synthesized Cu-based MOFs (NENU-5) with MoC_x_ nano-octahedral hydrogen evolution electrocatalysts with excellent HER activity. Recently, they used an ion exchange method to convert ${\text{MO}}_{4}^{2-}$ groups by employing an exchange reaction between a zinc-based imidazole MOF (ZIF-8) and a metal salt (Na_2_MO_4_, M=Mo or W) in an organic solvent (Lu et al., 2019) (Figure 1B). The ${\text{MO}}_{4}^{2-}$ group replaces the ${\text{Zn}(\text{imidazolate})}_{4}^{2-}$ group in ZIF-8, thereby effectively dispersing and fixing the metal source uniformly in the ZIF-8 framework (Mo_x_C/PNCDs). Due to the structural advantages of ZIF-8, the low boiling point of Zn and the controlled exchange of ${\text{MO}}_{4}^{2-}$, ultrafine carbide nanocrystals with a porous nitrogen-doped carbon dodecahedron were successfully obtained. And achieve effective control of the carbide phase and composition, which not only provides a more stable active site, but also promotes electron transport during the HER process.

### Secondary Conversion of Organic-Inorganic Hybrid Materials (SC-OI-M)

Currently, there are few simple and diverse synthesis methods for new MOF materials, which greatly limits their development and application. In addition, conventional MOFs only have micropores and lack transmission channels such as mesopores and macropores, which will greatly reduce their transmission efficiency in the catalytic process. SC-OI-M refers to the integration of two counterparts into a single structure at the nanoscale (Wang et al., 2018b; Li et al., 2019a). This nano-scale single structure provides a periodic organic-inorganic structure, which has a “barrier effect” between the Mo sources during the high-temperature carbonization process, which can effectively prevent and promote the formation of nanostructures during high-temperature sintering. The hybrid materials generate a large amount of reducing gases such as CH_x_, CO, and H_2_ under high temperature decomposition to achieve the *in-situ* conversion of molybdenum carbide. The structure, morphology and composition of the synthesized molybdenum carbide have excellent controllability. Liao et al. (2014) synthesized nanoporous Mo_2_C Nanowires (np-Mo_2_C NWs) by pyrolyzing MoO_x_/amine hybrid precursors with sub-nanometer periodic structure. This became the beginning of the “SC-OI-M” for the development of highly active hydrogen evolution catalysts. β-Mo_2_C has outstanding HER performance, but it often exhibits excessive hydrogen absorption capacity, which restricts the desorption step of hydrogen atoms in HER (i.e., Heyrovsky/Tafel) (Wan et al., 2014). Shi et al. (2017) used electrostatic assembly to prepare a three-component hybrid precursor of MoO_x_-phytic acid-polyaniline. P-doped β-Mo_2_C composite nanowire electrocatalyst (P-Mo_2_C@C NWs) was obtained after high temperature carbonization (Figure 1C). On the nanometer scale, the uniform ultra-small β-Mo_2_C particles provide abundant exposed catalytically active sites, and the structure of the one-dimensional nanowire facilitates the radial conduction of electrons. The resulting graphitized carbon greatly improves the overall conductivity and stability of the catalyst. At the atomic scale, the introduction of P atoms effectively increases the electron cloud density of the β-Mo_2_C Fermi level, and introduces steric hindrance effects, effectively weakens the Mo-H bond, reduces the ΔGH^*^ of β-Mo_2_C. Experimental and theoretical calculations show that suitable P doping (2.9%) can effectively balance the Volmer and Heyrovsky/Tafel processes in the HER and optimize the intrinsic activity of β-Mo_2_C for hydrogen evolution. Based on the controllability of this method, the researcher also proposed a series of electronic and spatial structure control methods. The formation of heterostructures such as by Co atom doping (Lin et al., 2016a), MoC-Mo_2_C (Lin et al., 2016b), and Fe_3_C-Mo_2_C (Lin et al., 2017a), the fine control of the surface and structure of β-Mo_2_C was realized, and the electrocatalytic performance was optimized.

## Regulate the Catalytic Performance of HP-Mo_x_C Based Catalysts

The key to construct a high-activity catalyst is using the advantages of HP-Mo_x_C catalyst with high electronic conductivity and large specific surface area, combined with the regulation of active sites. There are two basic principles in the regulation of catalyst performance namely increasing the active site and enhancing the intrinsic activity of the material. For the former, it can be achieved by porous structure; for the latter, it can be achieved by heteroatom doping and constructing heterostructure.

### Introducing Other Conductive Carriers

Enriched active sites of sufficient unsaturated Mo and C atoms promote intimate contact between the electrolyte and the electrode material, thereby enhancing catalytic performance (Wang et al., 2019a; Cui et al., 2020). The introduction of a suitable matrix to form a strong coupling toward the catalyst can improve the intrinsic catalytic activity of Mo_x_C, and also increase the conductivity of the catalyst due to the synergistic effect in the hybrid nanostructure, and hance, finally harvest desired electrochemical performance (Amrute1 et al., 2019; Xiong et al., 2019; Zhao et al., 2019c).

Up to now, a series of carbon materials such as reduced graphene oxide and carbon nanotubes have been used for supporting molybdenum carbide particles because of their large surface area and excellent electronic conductivity (Huang et al., 2019c; Li et al., 2019d; Wang et al., 2019e; Yu et al., 2019). Pan et al. (2014) used glucose as GO stabilizer, carbon source and reducing agent to prepare Mo_2_C nanoparticles grown on reduced graphene oxide (Mo_2_C-RGO), which showed excellent HER electrocatalytic activity. Huang et al. (2019c) developed a new technology for microwave-assisted ultrafast preparation of high performance carbon supported molybdenum carbide catalysts (Mo_2_C/G) (Figure 1D). They impregnated the carbon support with ammonium molybdate solution and irradiated it with microwave to obtain a carbon-supported molybdenum carbide catalyst. The carbon carrier material acts as an absorbing medium to promote *in-situ* rapid heating of the material, and a carbon source for forming Mo_2_C. This method can be applied to a variety of large-scale production of carbon carriers, including graphene, carbon nanotubes, commercial carbon black, and carbon fiber, etc. The *in situ* formation of the carbon support ensures a tight interfacial contact between the active material and the conductive substrate, thereby promoting rapid charge transfer between the substrate and the active material (Zhang et al., 2018; Han et al., 2019; Hou et al., 2019). Finding a way to synthesize molybdenum carbide/carbon support composites in one step is very valuable (Hsieha et al., 2019; Wu et al., 2019). Cheng et al. (2018) synthesized a 1 nm-sized molybdenum carbide nanoparticle in a carbon (Mo_2_C@NC) nanomesh through hydrothermal treatment, using dicyandiamide as a carbon and nitrogen source, ammonium molybdate as a molybdenum source. During the hydrothermal process, the intermolecular hydrogen bonding is used to self-assemble into band structure to limit the growth of particles. This mesoporous ribbon nanoweb structure provides a high specific surface area and a rich active site, greatly reduced the energy barrier. This material has excellent HER/ORR performance compared to other materials. This work demonstrates a simple template-free strategy for the synthesis of highly efficient non-precious metal catalysts with large specific surface areas. It also shows the possibility of replacing platinum-based catalysts with molybdenum carbide materials from theoretical to experimental evidence.

### Doping

The performance of the catalyst can also be controlled by doping. Heteroatom doping can be combined with addition of conductive support to enhance the intrinsic activity of the materials besides increasing the active site of the catalysts. The doping of heteroatoms into the lattice of the catalyst can adjust the electron and surface structure of the material, thereby affecting the adsorption free energy of the reaction intermediate on the surface, and improving the catalytic efficiency (Jia et al., 2017; Guo et al., 2019; Li et al., 2019a). At present, non-metal atoms (N, S, P, B, etc.), transition metals (Fe, Co, Ni, Zn, etc.) are introduced to replace Mo/C atoms in Mo_x_C. It can adjust the intrinsic electron configuration of molybdenum carbide and improve the conductivity (Li et al., 2019c; Wang et al., 2019c,f; Zhong et al., 2019).

Nitrogen-doped nano-carbon support plays an important role in improving electrocatalytic activity (Wu et al., 2018a; Lyu et al., 2019). Wu et al. (2016) synthesized ultrafine Mo_2_C nanoparticles embedded within bacterial cellulose-derived 3D N-doped carbon nanofiber networks (Mo_2_C@N-CNFs). Theoretical calculations demonstrate that excellent HER activity results from a strong synergistic effect between Mo_2_C nanocatalyst and N-CNF. Transition metal dopants (Ni, Co, Fe, etc.) can improve catalytic performance by adjusting the electronic configuration, creating new active sites and activating surrounding sites (Wu et al., 2018b; Cao et al., 2019). Li et al. (2019b) synthesized Ni/Mo_2_C nitrogen-doped carbon nanofibers (Ni/Mo_2_C-NCNFs) by using the electrospinning method (Figure 1E). Synergistic effect between Ni and Mo_2_C nanoparticles, high conductivity, large electrochemical active surface area and effective N doping significantly promote HER and OER due to strong hydrogen binding energy on Mo_2_C and high conductivity of Ni. This work provides a facile and effective way to produce low cost and high performance dual functional electrocatalysts for efficient overall water splitting. Since a single heteroatom doping has been shown to improve the catalytic performance of carbon materials, researchers developed binary or multi-heteroatom doped carbon materials to adjust the d-orbitals and optimize the electronic structure (Du et al., 2019; Ling et al., 2019; Kou et al., 2020). Ang et al. (2016) reported the formation of layered Mo_2_C by carburizing of molybdenum oxide/phenol/thioacetamide hybrids, followed by solvent stripping of layered Mo_2_C to further form N/S co-doped molybdenum carbide nanosheets. The synthesized nanosheet has an ultrathin thickness (1 nm) and a large specific surface area (139 m^2^ g^−1^). The incorporated N and S effectively improve the wettability of the material. Wang et al. (2018a) synthesized NP-Mo_2_C by direct carbonization. Theoretical calculations show that the doping of heteroatoms into carbon promotes the transfer of electrons in the catalyst, and the heteroatoms may also act as catalytic active sites (Gao et al., 2019; Singh et al., 2019). Owing to the synergistic coupling, the double doping of the N, P heteroatoms in Mo_2_C can remarkably improve the intrinsic activity of each active site.

### Hierarchical Porous Molybdenum Carbide-Based Heterostructure

Heterostructured hybrids have shown superior electrochemical performance compared to the corresponding single components (Liang et al., 2019; Yao et al., 2019). Through the interface electron transfer in a heterostructure, a large number of interfaces between multiple components can induce optimization of the electronic configuration (Wang et al., 2019d; He et al., 2020). Therefore, constructing heterojunction is an effective way to optimize the free energy of hydrogen adsorption.

The engineering design of molybdenum carbide-based heterostructures provides a new perspective for electrocatalysis. For example, heterostructures of Mo-Mo_2_C (Dong et al., 2018), Mo_2_C-Mo_2_N (Chen et al., 2013), MoP@Mo_2_C (Huang et al., 2018), and (Mo_2_C)_x_-(WC)_1−x_-QDs (Huo et al., 2017). The synergistic effect of the interface portion exhibits superior HER performance over the single component molybdenum carbide. Dong et al. (2018) obtained a new Mo-rich molybdenum carbide-based electrocatalyst (Mo-Mo_2_C) by calcining Mo_3_O_10_(C_6_H_8_N)_2_∙2H_2_O precursor (Figure 1F). The overpotential of the Mo-Mo_2_C hydrogen evolution reaction is only 67 mV, and the Tafel slope is as low as 55 mV/dec. Its excellent HER performance can be attributed to the improvement of the internal charge transfer ability of the catalyst.

Recently, Huang et al. (2019a) constructed a Mo_2_C/VC heterostructure. The CO_2_ decomposed from Mg and NaHCO_3_ reacts at high temperature to generate a three-dimensional carbon network. At the same time, the micro-scale precursor V_2_MoO_8_ is broken into two-phase materials Mo_2_C and VC that are not completely separated and embedded in the three-dimensional carbon network. The three-dimensional conductive carbon network and the cross-linked structure fully provide electron transportability and structural stability. Mo_2_C has strong hydrogen adsorption capacity, while VC has strong hydrogen desorption capacity, so single component Mo_2_C@C and VC@C exhibit poor hydrogen evolution reactivity, while Mo_2_C/VC@C shows rapid adsorption capacity and rapid desorption kinetics. Based on the combination of experiment and theory, this experiment proposes that the method of preparing a rich interface structure by phase separation is a way to efficiently prepare high performance catalyst. This approach can be extended to other highly efficient heterogeneous catalysts and different energy sources in the future.

## Discussion

In summary, molybdenum carbide-based materials are an ideal HER material. In this review, recent developments in the structural design and electronic regulation of molybdenum-based catalysts are illustrated. Template method, morphology-conserved transformations method, and secondary conversion of organic-inorganic hybrid materials method are effective strategies for synthesizing various molybdenum carbide-based materials. By compounding with a conductive carrier, element doping and designing a heterojunction can achieve electronic optimization of HER kinetics, greatly improving catalyst activity and stability. In practical applications, material combinations and properties, flexible selection of synthesis methods and performance control methods can synergistically achieve highly efficient catalysts. However, the large-scale application of electrolyzed water for hydrogen evolution still has a long way to go. In combination with the rapid development of molybdenum carbide in electrocatalytic hydrogen evolution in recent years. Future research on molybdenum carbide catalysts may focus on the development of efficient new synthetic methods, the development of molybdenum carbide hydrogen evolution devices, mechanism research, standardized test and the mining and understanding of structure-activity relationships.

## Author Contributions

All authors listed have made a substantial, direct and intellectual contribution to the work, and approved it for publication.

## Conflict of Interest

The authors declare that the research was conducted in the absence of any commercial or financial relationships that could be construed as a potential conflict of interest.
